# Breed susceptibility for common surgically treated orthopaedic diseases in 12 dog breeds

**DOI:** 10.1186/s13028-019-0454-4

**Published:** 2019-04-25

**Authors:** Gudrun Seeberg Boge, Elena Regine Moldal, Maria Dimopoulou, Eystein Skjerve, Annika Bergström

**Affiliations:** 1 Department of Companion Animal Clinical Sciences, Faculty of Veterinary Medicine, Norwegian University of Life Sciences, P.O. Box 369 sentrum, N-0102 Oslo, Norway; 2 Department of Clinical Sciences, Swedish University of Agricultural Sciences, P.O. Box 7054, SE-750 07 Uppsala, Sweden; 3 Department of Food Safety and Infection Biology, Faculty of Veterinary Medicine, Norwegian University of Life Sciences, P.O. Box 369 sentrum, N-0102 Oslo, Norway

**Keywords:** Canine, Cruciate ligament, Elbow dysplasia, Fractures, Patellar luxation, Radius, Ulna

## Abstract

**Background:**

A retrospective case–control study was conducted to estimate breed predisposition for common orthopaedic conditions in 12 popular dog breeds in Norway and Sweden. Orthopaedic conditions investigated were elbow dysplasia (ED); cranial cruciate ligament disease (CCLD); medial patellar luxation (MPL); and fractures of the radius and ulna. Dogs surgically treated for the conditions above at the Swedish and Norwegian University Animal Hospitals between the years 2011 and 2015 were compared with a geographically adjusted control group calculated from the national ID-registries. Logistic regression analyses (stratified for clinic and combined) were used to calculate odds ratios (OR) and 95% confidence intervals. Mixed breed dogs were used as reference.

**Results:**

Breeds found at-risk for ED were the Labrador retriever (OR = 5.73), the Rottweiler (OR = 5.63), the German shepherd dog (OR = 3.31) and the Staffordshire bull terrier (OR = 3.08). The Chihuahua was the only breed where an increased risk for MPL (OR = 2.80) was identified. While the Rottweiler was the only breed predisposed for CCLD (OR = 3.96), the results were conflicting for the Labrador retriever (OR = 0.44 in Sweden, 2.85 in Norway); the overall risk was identical to mixed-breed dogs.

**Conclusions:**

Most results are in concordance with earlier studies. However, an increased risk of CCLD was not identified for the Labrador retriever, the Staffordshire bull terrier was found to have an increased risk of ED and some country-specific differences were noted. These results highlight the importance of utilising large caseloads and appropriate control groups when breed susceptibility is reported.

**Electronic supplementary material:**

The online version of this article (10.1186/s13028-019-0454-4) contains supplementary material, which is available to authorized users.

## Background

Surgical correction of orthopaedic disease implies pain and sometimes an uncertain prognosis for the animal, in addition to emotional stress for both the dog and its owner. Moreover, the time and money spent on veterinary consultations and an often extensive postoperative rehabilitation process should not be neglected. Information regarding breed susceptibility in different orthopaedic disorders in dogs may aid in the development of preventive measures, as well as act as a guide for potential pet owners and a motivational measure for dog breeders.

Most of the common orthopaedic diseases seen in small animal practice today are considered multifactorial in origin, with physical conformation and genetics being predisposing factors. Several epidemiological studies have reported the prevalence of different orthopaedic conditions and their risk factors in dogs, including breed predisposition. Most of these studies have sampled the study subjects, both cases and controls, from hospital populations, often at larger referral and university hospitals, and have not taken the breed distribution of the background population into account [1, 2].

The purpose of controls is to provide valid information regarding the background frequency of an exposure (i.e. a particular dog breed) within the population at risk of becoming a case (i.e. individuals who are free of the disease in question) [3–5]. Correct control selection is crucial to the internal validity of case–control studies [6]. When both cases and controls are collected from hospital populations in defined geographic areas, the controls may fail to provide an unbiased sample of the population at risk, and results in respect to exposure status might be unreliable [3, 5]. In the context of breed susceptibility this may lead to an incorrect impression that some popular breeds are predisposed to conditions when in fact they are not. Hence, it is not surprising that the reported breed predispositions differ between studies [1, 2].

Unaffected individuals from the population of animals in the same geographic region as the hospitals where the cases are collected, can be used as controls to enhance the probability that cases and controls come from the same source population [7]. In Norway and Sweden, comprehensive national ID-registries containing searchable information of all ID-marked dogs (DyreID and DjurID, respectively) are available. ID-marking (microchipping) is mandatory for all dogs holding a passport in Europe,^1^ all dogs in Sweden,^2^ as well as for pure-breed dogs registered in the Norwegian Kennel Club.^3^ Even though ID-marking is not mandatory for mixed-breed dogs in Norway, it is estimated that approximately 85% of all Norwegian dogs are marked (Vatn G, personal communication 2018). The numbers are likely higher in Sweden. The ID-databases provide an opportunity for selection of control animals from the same geographical areas as the hospital populations, and thereby increase the likelihood of sampling controls from the same source population as the cases.

The objective of this study was to estimate breed susceptibility for common orthopaedic conditions in popular dog breeds in Norway and Sweden.

## Methods

### Study design

A retrospective case–control study was performed, utilising clinical, demographic and geographic data from two Veterinary Teaching Hospitals in Norway and Sweden and demographic and geographic data from the Norwegian and Swedish national ID-registries, DyreID and DjurID.

### Data extraction and study population

The study population consisted of all canine patients treated at two Veterinary Teaching Hospitals (VTH); University Animal Hospital, Swedish University of Agricultural Sciences (SLU) and University Animal Hospital, Norwegian University of Life Sciences (NMBU), between January 1, 2011 through December 31, 2015. Cases were purposively sampled from the study population to ensure inclusion of the most common surgically treated orthopaedic diseases and common dog breeds in the source population. Medical records of all dogs that were surgically treated for orthopaedic diseases were reviewed retrospectively and registered in a database. Diagnosis, demographic (breed, age, sex, body weight) and geographic (VTH and dog owners’ county of residence at the time of surgery) data were recorded and each record was screened for completeness. Only initial surgery was recorded for animals with bilateral disease. Dogs were eligible for inclusion if they had a confirmed primary orthopaedic diagnosis in the medical records. For example, a diagnosis of medial patellar luxation (MPL) secondary to trauma with multiple injuries was excluded.

The national ID-databases in Norway and Sweden were chosen for generation of an appropriate control group. For the control group to be comparable to the study population in respect to demographic factors, the search was limited to dogs born between 2006 and 2015. To ensure inclusion of the most abundant breeds in the geographical areas where the study population originated, only dogs belonging to the 50 most common breeds in each of the Norwegian and Swedish counties were collected from the national ID-databases. The Fédération Cynologique Internationale (FCI) classification was used for breed classification.

### Data handling

Substantial data cleaning steps were undertaken to ensure selection of the most commonly represented breeds in the source population, and that the eligibility criteria were met in such a way that the case and control populations were comparable.

First, the geographical distribution of dogs surgically treated for orthopaedic diseases in the study population was calculated separately for each country to estimate the geographical distribution of the source population. The dogs eligible for inclusion came from 17/21 Swedish and 16/18 Norwegian counties. The number of dogs from each county was divided by the total number of eligible dogs and reported as a percentage. The numbers from counties with less than 1% of the cases in the database (< 5 cases) were excluded to avoid overemphasising the importance of counties with a marginal contribution to the study population. Seven Swedish and nine Norwegian counties contributed with more than 1% each and were included in the calculations. The relative contributions of the 16 counties retained are given in Table 1.

**Table 1:** Geographical distribution of dogs surgically treated for orthopaedic diseases at two Veterinary Teaching Hospitals

Swedish county	N (%)	Norwegian county	N (%)
Gävleborg	68 (14.47)	Akershus	107 (23.11)
Norrbotten	5 (1.06)	Buskerud	40 (8.64)
Stockholm	118 (25.11)	Hedmark	37 (7.99)
Uppsala	257 (54.68)	Oppland	16 (3.46)
Västerbotten	5 (1.06)	Oslo	208 (44.92)
Västernorrland	6 (1.28)	Telemark	28 (6.05)
Västmanland	11 (2.34)	Trøndelag	6 (1.30)
		Vestfold	15 (3.24)
		Østfold	6 (1.30)
Total included	470 (94.76)		463 (94.88)
Other counties^a^	26 (5.24)		25 (5.12)
Total	496 (100.00)		488 (100.00)

Data presented as number (percentage) of dogs surgically treated for orthopaedic diseases at the University Animal Hospital, Swedish University of Agricultural Sciences and the University Animal Hospital, Norwegian University of Life Sciences over a 5-year period

^a^10 Swedish and 7 Norwegian counties < 1% of caseload not included in control group calculations

**Fig. 1:**
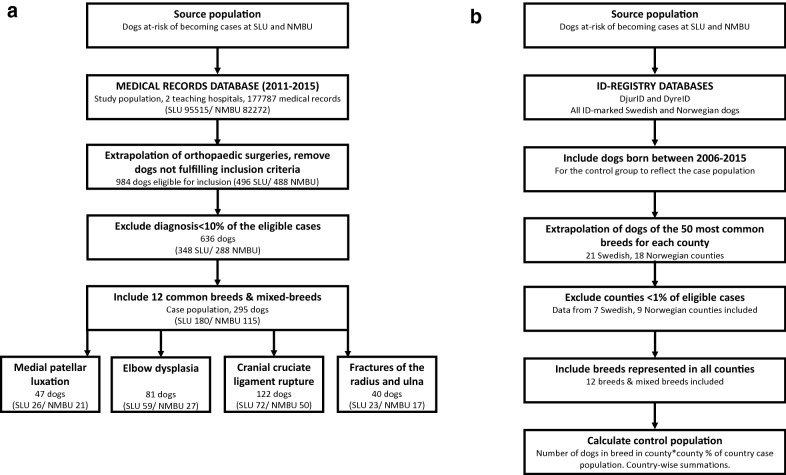
Schematic illustration of the case- and control population selection. **a** Case population, **b** control population

Second, to select the most common surgically treated orthopaedic diseases and ensure statistical reliability, dogs with diagnoses with less than 100 individual recordings (comprising < 10% of the eligible cases) were excluded. Four diagnoses included more than 100 recordings; medial compartment disease (MCD), fractures of the radius/ulna, MPL and cranial cruciate ligament disease (CCLD). Since MCD is closely associated with the other developmental elbow joint diseases, we chose to also include humeral trochlear osteochondrosis (OC) and ununited anconeal process (UAP) in one combined elbow dysplasia (ED) category. These four conditions are further referred to as the diseases under study (Fig. 1a).

Third, the control group retracted from the ID-registries was restricted to breeds present in all the counties selected in the first step (Fig. 1b). In addition to mixed-breed dogs, the Border Collie, Cavalier king Charles spaniel (CKCS), Chihuahua, English cocker spaniel, Flat-coated retriever, German shepherd dog (GSD), Golden retriever, Jack Russell terrier (JRT), Labrador retriever, Rottweiler, Shetland sheepdog, and the Staffordshire bull terrier were among the 50 most common breeds in all counties. These breeds are further referred to as the breeds under study.

Fourth, the control group retracted from the ID-registries was modified to reflect the breed distribution in the source population. The number of dogs in each of the breeds under study in the included counties were adjusted in accordance with the percentage of the eligible cases in the study population from the respective county (given in Table 1). While 55% of the eligible cases at SLU were from the county of Uppsala, 25% were from Stockholm, but only 1% from Västerbotten. In the national ID-registry, 755 Labradors were registered in Uppsala, 3331 in Stockholm and 452 in Västerbotten. These numbers where then multiplied (755 * 0.55 = 415, 3331 * 0.25 = 833 and 452 * 0.01 = 5) and similar calculations were performed for the other counties. Summed together the adjusted number of Labradors comprising the control population was 1277, which is closer to the raw registration numbers in Uppsala than in Stockholm. Calculations and raw registration numbers are provided separately (Additional file 1).

The final case population included all dogs in the study population of the breeds under study with the diagnosis of interests fulfilling the inclusion criteria.

### Statistical analysis

Data were complied, cleaned and checked for errors in Microsoft Excel and imported into Stata 14.2 (Stata Corp., College Station, TX, USA), which was used for all statistical analyses. Age and weight of all dogs with the diseases under study are presented as median (range). The case population for each diagnosis was regarded as a separate population for the statistical analysis. Univariable logistic regression was used to compare the breed distribution between the case- and control population separately for each country for the diagnoses under study with mixed-breed dogs as the reference. Breeds without cases of the diagnoses under study were omitted from the analysis. Multivariable logistic regression, with a fixed effect for VTH to adjust for country differences, was performed for the combined case population. Results are presented as odds ratios (OR) with 95% confidence intervals. As this was not a planned hypothesis testing study, no predefined level of significance is reported.

## Results

During the 5-year study period, a total of 82,272 individual patient records (average 16,455/year) were registered at NMBU and 95,515 (average 19,103/year) at SLU. Of these, 983 dogs (495 at SLU and 488 at NMBU) classified into 35 different diagnoses (Table 2), were eligible for inclusion in the study and 636 (64.6%) were treated for the diagnoses under study. ED, MPL and fractures of the radius/ulna occurred most frequently in young dogs, while CCLD had a median age of 5.8 years. ED and CCLD occurred most commonly in medium and large sized dogs, while the median weight for both MPL and fractures of the radius/ulna was below 5 kg (see Table 3 for more details).

**Table 2:** Distribution of orthopaedic disorders in surgically treated dogs at two Veterinary Teaching Hospitals

Disorder or injury	N (%)
Fracture tarsus	10 (1.02)
Infraspinatus contracture	11 (1.12)
Shoulder complex	15 (1.52)
Fracture MC/MT/Paw	19 (1.93)
OC Stifle	20 (2.03)
Luxation hip	21 (2.13)
Fracture humerus	22 (2.24)
Collateral ligament rupture	22 (2.24)
Fracture femur	34 (3.46)
Fracture tibia/fibula	48 (4.88)
OC Shoulder	52 (5.29)
Other diagnoses*	74 (7.52)
Total other diagnoses	348 (35.6)
Fracture radius/ulna	114 (11.59)
Elbow dysplasia	131 (13.31)
*MCD*	*103* (*10.47*)
*OC elbow*	*23* (*2.34*)
*UAP*	*5 (0.51)*
Medial patellar luxation	131 (13.31)
CCLD	260 (26.42)
Total diagnosis of interest	636 (64.6)
Total	984 (100)

Data presented as the number (percentage) of dogs surgically treated for orthopaedic diseases at the University Animal Hospital, Swedish University of Agricultural Sciences and the University Animal Hospital, Norwegian University of Life Sciences over a 5-year period

Italic is used to mark the diagnoses that is combined in the elbow dysplasia category

*OC* osteochondrosis, *UAP* ununited anconeal process, *MC* metacarpus, *MT* metatarsus, *MCD* medial compartment disease, *CCLD* cranial cruciate ligament disease

*Diagnoses with <1% of surgically treated orthopaedic cases summarised

**Table 3:** Age and body weight in relation to orthopaedic diagnosis at two Veterinary Teaching Hospitals

Disorder or injury	Age (years)	Weight (kg)
ED	1.0 (0.4–8.8)	30.0 (10.0–52.7)
*OC*	*0.9* (*0.5*–*8.4*)	*32.0* (*15.0*–*52.0*)
*MCD*	*1.0* (*0.4*–*8.8*)	*29.0* (*10.0*–*52.7*)
*UAP*	*0.5* (*0.4*–*2.2*)	*33.5* (*19.0*–*36.7*)
Medial patellar luxation	2.0 (0.6–8.9)	4.9 (1.6–27.0)
CCLD	5.8 (0.9–12.0)	26.2 (4.0–66.0)
Fracture of the radius/ulna	1.0 (0.2–8.0)	3.0 (1.0–37.8)

Data presented as median (range) and includes 984 dogs surgically treated for four common orthopaedic diseases at the University Animal Hospital, Swedish University of Agricultural Sciences and the University Animal Hospital, Norwegian University of Life Sciences over a 5-year period

Italic is used to mark the diagnoses that is combined in the elbow dysplasia category

*ED* elbow dysplasia, *OC* osteochondrosis, *UAP* ununited anconeal process, *MCD* medial compartment disease, *CCLD* cranial cruciate ligament disease

The breeds under study comprised 43.7% (430 dogs) of the eligible cases (Table 4), 51.2% in Sweden (254 dogs) and 36.1% in Norway (176 dogs). Sixty-eight percent (295 dogs) had one of the diagnoses in question and were included in the case population.

**Table 4:** Breed distribution of dogs surgically treated for orthopaedic diseases and a geographically adjusted control group

Breed	Eligible cases			Control population		
	Sweden	Norway	Combined	Sweden	Norway	Combined
	N (%)	N (%)	N (%)	N (%)	N (%)	N (%)
Mixed-breed	114 (44.9)	53 (30.1)	167 (38.8)	2964 (27.2)	4359 (37.4)	7323 (32.5)
Border collie	8 (3.2)	5 (2.8)	13 (3.0)	303 (2.8)	425 (3.7)	728 (3.2)
CKCS	5 (2.0)	9 (5.1)	14 (3.3)	522 (4.8)	556 (4.8)	1078 (4.8)
Chihuahua	14 (5.5)	18 (10.2)	32 (7.4)	1044 (9.6)	985 (8.5)	2029 (9.0)
English cocker spaniel	1 (0.4)	3 (1.7)	4 (0.9)	515 (4.7)	539 (4.6)	1054 (4.7)
Flat-coated retriever	3 (1.2)	4 (2.3)	7 (1.6)	433 (4.0)	356 (3.1)	789 (3.5)
German shepherd dog	12 (4.7)	7 (4.0)	19 (4.4)	1061 (9.7)	591 (5.1)	1652 (7.3)
Golden retriever	17 (6.7)	9 (5.1)	26 (6.1)	999 (9.2)	754 (6.5)	1753 (7.8)
Jack Russel terrier	9 (3.5)	6 (3.4)	15 (3.5)	430 (3.9)	710 (6.1)	1140 (5.1)
Labrador retriever	32 (12.6)	20 (11.4)	52 (12.1)	1277 (11.7)	823 (7.1)	2100 (9.3)
Rottweiler	19 (7.5)	22 (12.5)	41 (9.5)	502 (4.6)	469 (4.0)	971 (4.3)
Shetland sheepdog	8 (3.2)	7 (4.0)	15 (3.5)	475 (4.4)	319 (2.7)	794 (3.5)
Staff. bull terrier	12 (5.8)	13 (7.4)	25 (5.8)	394 (3.6)	763 (6.6)	1157 (5.1)
Total (BuS)*	254 (51.2)	176 (36.1)	430 (43.7)	10.525 (100.0)	10.886 (100.0)	21.411 (100.0)
Other breeds*	242 (48.8)	312 (63.9)	554 (56.3)			
Total	496 (100.0)	488 (100.0)	984 (100.0)			

Control population calculated from registration numbers of each breed in the national ID-registries adjusted to reflect the source population of dogs surgically treated for orthopaedic diseases at the University Animal Hospital, Swedish University of Agricultural Sciences and the University Animal Hospital, Norwegian University of Life Sciences over a 5-year period

Data presented as number of dogs (percentage of breeds under study)

*BuS* Breeds under study, *CKCS* Cavalier king Charles spaniel

*Data presented as number of dogs (percentage of total)

Details from the logistic regression analyses including OR, confidence intervals and associated P-values are given in Table 5. The German shepherd dog, Labrador retriever, Rottweiler and the Staffordshire bull terrier were identified with an increased risk of ED (Table 5a). The highest risk was found for the Labrador (OR = 5.73) and Rottweiler (OR = 5.63). The Chihuahua was the only breed with an increased risk of MPL (OR = 2.80, Table 5c). Together with the GSD, the Chihuahua was also found to have a decreased risk of CCLD (Table 5b). The risk of CCLD in the Labrador retriever was lower than for mixed-breed dogs in Sweden (OR = 0.44), but higher in Norway (OR = 2.85) and the combined analysis gave an OR equal to mixed-breed dogs. The Rottweiler was the only breed where an increased risk of CCLD was identified (OR = 3.96). In addition to mixed-breeds, only three of the breeds under study (the CKCS, Chihuahua and the Shetland sheepdog) had cases of fractures of the radius and ulna (Table 5d), but no difference in risk could be identified. The OR for being treated for the diseases of interests were generally lower at NMBU compared to SLU (OR 0.50–0.67).

**Table 5:** Results from the logistic regression analyses of breed susceptibility for four common orthopaedic diseases in 12 dog breeds

a) Elbow dysplasia												
Breed	SLU				NMBU				Combined			
	N (%)	OR	95% CI	P	N (%)	OR	95% CI	P	N (%)	OR	95% CI	P
Mixed-breed	13 (22.0)	1.00	Ref.	–	2 (7.41)	1.00	Ref.	–	15 (17.4)	1.00	Ref.	–
Flat-coated retriever	0 (0.0)	–	–	–	2 (7.41)	12.24	(1.72–87.18)	0.012	2 (2.3)	1.13	(0.26–4.96)	0.872
German shepherd dog	10 (17.0)	2.15	(0.94–4.92)	0.070	3 (11.11)	11.06	(1.84–66.35)	0.009	13 (15.1)	3.31	(1.56–7.02)	0.002
Golden retriever	7 (11.9)	1.60	(0.63–4.02)	0.319	2 (7.41)	5.78	(0.81–41.10)	0.080	9 (10.5)	2.26	(0.98–5.18)	0.055
Jack Russel terrier	0 (0.0)	–	–	–	0 (0.0)	–	–	–	0 (0.0)	–	–	–
Labrador retriever	19 (32.2)	3.39	(1.67–6.89)	0.001	9 (33.33)	23.83	(5.14–110.51)	< 0.001	28 (32.6)	5.73	(3.04–10.81)	< 0.001
Rottweiler	4 (6.8)	1.81	(0.59–5.59)	0.298	8 (29.63)	37.18	(7.87–175.58)	< 0.001	12 (14.0)	5.63	(2.62–12.07)	< 0.001
Staff. bull terrier	6 (10.2)	3.47	(1.31–9.19)	0.012	1 (3.7)	2.86	(0.25–31.54)	0.392	7 (8.1)	3.08	(1.25–7.59)	0.014
SLU	59 (68.9)								59 (45.0)	1.00	Ref.	–
NMBU					27 (60.0)				27 (20.6)	0.52	(0.33–0.83)	0.006
Other breeds	27 (31.1)				18 (40.0)				45 (34.4)			
Total	86 (100.0)				45 (100.0)				131 (100.0)			

b) Cranial cruciate ligament disease												
Breed	SLU				NMBU				Combined			
	N (%)	OR	95% CI	P	N (%)	OR	95% CI	P	N (%)	OR	95% CI	P
Mixed-breed	32 (44.4)	1.00	Ref.	–	13 (26.0)	1.00	Ref.	–	45 (36.9)	1.00	Ref.	–
Border collie	0 (0.0)	–	–	–	1 (2.0)	0.79	(0.10–6.05)	0.820	1 (0.8)	0.22	(0.03–1.61)	0.136
CKCS	0 (0.0)	–	–	–	2 (4.0)	1.21	(0.27–5.36)	0.805	2 (1.6)	0.30	(0.07–1.23)	0.096
Chihuahua	1 (1.39)	0.09	(0.01–0.65)	0.017	1 (2.0)	0.34	(0.04–2.61)	0.299	2 (1.6)	0.16	(0.04–0.66)	0.011
English cocker spaniel	0 (0.0)	–	–	–	1 (2.0)	0.62	(0.08–4.76)	0.648	1 (0.8)	0.15	(0.02–1.11)	0.064
German shepherd dog	0 (0.0)	–	–	–	1 (2.0)	0.57	(0.07–4.34)	0.585	1 (0.8)	0.10	(0.01–0.70)	0.021
Golden retriever	7 (9.7)	0.65	(0.29–1.48)	0.302	5 (10.0)	2.22	(2.22–1.17)	0.130	12 (9.8)	1.09	(0.57–2.07)	0.796
Jack Russel terrier	7 (9.7)	1.51	(0.66–3.44)	0.329	2 (4.0)	0.94	(0.21–4.19)	0.940	9 (7.4)	1.29	(0.62–2.64)	0.495
Labrador retriever	6 (8.3)	0.44	(0.18–1.04)	0.062	7 (14.0)	2.85	(1.13–7.17)	0.026	13 (10.7)	1.00	(0.54–1.85)	0.991
Rottweiler	12 (16.7)	2.21	(1.13–4.33)	0.020	12 (24.0)	8.58	(3.89–18.91)	< 0.001	24 (19.7)	3.96	(2.39–6.56)	< 0.001
Shetland sheepdog	3 (4.17)	0.59	(0.18–1.92)	0.376	0 (0.0)	–	–	–	3 (2.5)	0.60	(0.19–1.95)	0.401
Staff. bull terrier	4 (5.6)	0.94	(0.33–2.67)	0.908	5 (10.0)	2.20	(0.78–6.18)	0.136	9 (7.4)	1.27	(0.62–2.62)	0.513
SLU	72 (50.3)								72 (27.7)	1.00	Ref.	–
NMBU					50 (42.7)				50 (19.2)	0.60	(0.42–0.87)	0.007
Other breeds	71 (49.7)				67 (57.3)				138 (53.1)			
Total	143 (100.0)				117 (100.0)				260 (100.0)			

c) Medial patellar luxation												
Breed	SLU				NMBU				Combined			
	N (%)	OR	95% CI	P	N (%)	OR	95% CI	P	N (%)	OR	95% CI	P
Mixed-breed	13 (50.0)	1.00	Ref.	–	8 (38.1)	1.00	Ref.	–	21 (44.7)	1.00	Ref.	–
CKCS	1 (3.9)	0.44	(0.06–3.35)	0.425	3 (14.3)	2.94	(0.78–11.11)	0.112	4 (8.2)	1.25	(0.43–3.66)	0.679
Chihuahua	9 (34.6)	1.97	(0.83–4.61)	0.120	8 (38.1)	4.43	(1.66–11.82)	0.003	17 (36.2)	2.80	(1.47–5.32)	0.002
Jack Russel terrier	1 (3.9)	0.53	(0.07–4.06)	0.248	0 (0.0)	–	–	–	1 (2.1)	0.31	(0.42–2.30)	0.252
Shetland sheepdog	2 (7.7)	0.96	(0.21–4.27)	0.957	0 (0.0)	–	–	–	2 (4.3)	0.81	(0.19–3.49)	0.782
Staff. bull terrier	0 (0.0)	–	–	–	2 (9.5)	1.43	(0.30–6.74)	0.652	2 (4.3)	0.62	(0.14–2.65)	0.518
SLU	26 (36.1)								26 (19.8)	1.00	Ref.	–
NMBU					21 (35.6)				21 (16.0)	0.67	(0.38–1.20)	0.181
Other breeds	46 (63.9)				38 (64.4)				84 (64.1)			
Total	72 (100.0)				59 (100.0)				131 (100.0)			

d) Fractures of the radius and ulna												
Breed	SLU				NMBU				Combined			
	N (%)	OR	95% CI	P	N (%)	OR	95% CI	P	N (%)	OR	95% CI	P
Mixed-breed	19 (82.6)	1.00	Ref.	–	9 (52.9)	1.00	Ref.	–	28 (70.0)	1.00	Ref.	–
CKCS	1 (4.4)	0.30	(0.04–2.24)	0.240	0 (0.0)	–	–	–	1 (2.5)	0.23	(0.32–1.71)	0.152
Chihuahua	3 (13.0)	0.45	(0.13–1.52)	0.197	6 (35.3)	2.95	(1.05–8.31)	0.041	9 (22.5)	1.09	(0.51–2.33)	0.816
Shetland sheepdog	0 (0.0)	–	–	–	2 (11.8)	3.04	(0.65–14.11)	0.156	2 (5.0)	0.59	(0.14–2.51)	0.480
SLU	23 (48.9)								23 (20.2)	1.00	Ref.	–
NMBU					17 (25.4)				17 (14.9)	0.58	(0.30–1.09)	0.090
Other breeds	24 (51.1)				50 (74.6)				74 (64.9)			
Total	47 (100.0)				67 (100.0)				114 (100.0)			

Results from country-stratified and combined logistic regression analyses presented as Odds ratios (OR) and 95% confidence intervals (CIs). Breeds without cases of the disease in question were omitted

*CKCS* Cavalier king Charles spaniel, *SLU* Swedish University of Agricultural Sciences, *NMBU* Norwegian University of Life Sciences, *ref* reference category

## Discussion

Three of the four breeds identified in this study as having an increased risk of surgery for ED are the same as in several other studies. The German shepherd dog, Labrador retriever, and the Rottweiler are well-known breeds at risk [8–11]. An interesting finding is that the Staffordshire bull terrier had a high OR for ED. To the authors’ knowledge, there is only one other study available reporting this breed among breeds predisposed for ED [9]. In Scandinavia, the Staffordshire bull terrier has gained great popularity over recent years and from being a rare breed has now become one of the most common breeds in both Norway and Sweden.^4^^,^^5^ If this is true also for other countries, it may help explain why this is the only other study to date concerning this breeds’ predisposition to ED. The Staffordshire bull terrier shares a common ancestry with Mastiff breeds, which are reported to have the disease [10].

It should be mentioned that the collective diagnosis ED used in this study comprises three common developmental disorders in the dog, UAP, MCP, and OC. Joint incongruity and articular cartilage damage are also included in the group of conditions known as elbow dysplasia^6^ but have not been evaluated in our study. However, since all conditions sorted under the collective term are believed to be highly interrelated [12] and articular cartilage damage and joint incongruity are unlikely to be seen as a separate entity, we believe this to be a minor limitation to the study. Moreover, conclusions about prevalence of the particular diagnoses in each breed has been addressed in previous studies [8–10].

Labrador retrievers, Rottweilers and Staffordshire bull terriers are reported to be at increased risk for CCLD, while Chihuahuas, GSDs, and Shetland sheepdogs have been claimed to be at lower risk [1, 2, 13–15]. Our study detected an increased risk of disease in the Rottweiler, and decreased in GSDs and Chihuahuas, which are consistent with the earlier reports. For some breeds the literature provides inconsistent results. Cocker spaniels were found to have a decreased risk of CCLD in one study [1], but not in another [15]. The risk among Golden retrievers have been described both as increased [1], same as in the reference population [14] and decreased [2, 15]. Despite the Labrador retriever being one of the most common breeds presenting with CCLD in our material, the combined OR was identical to mixed-breed dogs. Though mixed-breeds have been reported to have a slightly higher OR for CCLD than purebred dogs [8], this finding highlights the importance of having a comparable control population when reporting breed susceptibility. The country-specific OR for CCLD in the Labrador was lower than for mixed-breed dogs in Sweden, but higher in Norway. As for several other breeds originally bred for hunting, and the retriever breeds in particular, there are two quite different types of Labradors; a slim, lighter working type and a heavier built show type. It is not known whether the likelihood of orthopaedic diseases is the same for both types. Moreover, the relative frequencies of show and field bred Labradors in Norway and Sweden are unknown. This could be a contributing factor to the deviating results observed in the two countries and illustrates that breed susceptibility reported from single-centre studies and/or studies with a limited caseload should not be overemphasised. In general, minimally/borderline significant results in relation to breed susceptibility should be viewed with caution.

Medial patellar luxation is far more common than lateral luxation [16]. Among the breeds reported to have a higher prevalence are the CKCS, JRT and the Chihuahua [10, 16–18]. The results are conflicting for Staffordshire bull terriers [17, 18]. Even though the CKCS had a slightly higher OR than mixed-breed dogs in our study, the Chihuahua was the only breed where an increased risk of surgically treated MPL was identified. This is in concordance with a recent study reporting the prevalence of patellar luxation among Swedish Chihuahuas to be 23% [19]. The Labrador retriever is reported with an increased prevalence of MPL in some studies [17, 20, 21], but Labrador retriever is also the most common purebred dog registered in the UK Kennel Club [22]. Two of the aforementioned studies were conducted in the UK, but since neither included a comparable control population, no conclusions about breed predispositions in the source population should be drawn. Even though the Labrador retriever is one of the most popular breeds in Norway and Sweden as well, no Labrador retrievers presented with MPL in our material. It may therefore seem that Scandinavian Labrador retrievers have a decreased rather than increased risk of MPL.

Considering the low bodyweight of the dogs with fractures of the radius and ulna in our material (Table 3), it is not surprising that the Chihuahua, CKCS and the Shetland sheepdog were the only breeds under study with the diagnosis. The absence of fractures of the radius and ulna in larger breeds was expected since these are more common in small and miniature dogs [23, 24].

The discrepancy between earlier studies and our results could be attributed to several factors such as genetic variation between different geographical areas and genetic drift as a consequence of breeding strategies over time [22], but it could also be due to the lack of an appropriate control group in previously published studies. In addition, a change of breed popularity over time, as discussed for the Staffordshire bull terrier, needs to be taken into account. Breed predispositions reported in studies conducted decades ago should be viewed with caution since they are likely to lack validity today. Comparing breed susceptibility with a control population adjusted to match the geographical distribution of the case population could be extended to larger caseloads from different geographical regions to increase the external validity of the results and to be able to calculate odds ratios for breeds where the diagnosis of interest is rare. A larger case population would improve the accuracy of the estimations and make it a better tool to study breeds with decreased risks, without the need for more advanced statistical methods. The method described in our study provides a framework with a potential for exploring breed-specific disease predispositions further. It is not limited to orthopaedic disorders but could be extended to all diseases where breed predisposition is suspected.

Most studies that report breed predispositions acknowledge the lack of a representative control population as a limitation. The control group is often either completely missing with only raw prevalence being described or limited to randomly selected hospital controls. Hospital populations, in particular referral populations, are mostly composed of sick dogs. Since sick dogs can acquire a different condition of interest, the dogs being sick is not in itself a justified reason for excluding them as controls. However, a variety of different diseases in dogs are breed-related. This introduces selection bias since some breeds are likely to be overrepresented in a study population comprised of sick dogs, and hospital populations are therefore not the most representative population for control selection in regard to breed composition. A source population is defined as the population from which the study subjects are drawn [6]. In some cases, the source population is well-defined, but more often, as in the case of hospital populations, where some animals might come from afar, while others live nearby, the actual source population from which the cases originate is unknown [4]. Some studies have utilised larger clinical databases, such as the VetCompass system in the UK [1] and the Veterinary Medical Databases in the USA [15]. Although these databases include large numbers of animals, they only contain information about dogs admitted to veterinary care, and not the actual source population (the population of dogs that were likely to be included as cases if they had got the disease in question). Even when large clinical databases are used, the reported risk of disease can appear too high if the breed under investigation has a lower than average disposition for other diseases, and therefore is less frequently represented in the clinical database than in the source population. In recent years, a Swedish database of insured dogs has been used to compare breed predisposition to different diseases [25–27]. A limitation of using insured dogs as the reference is that the uninsured dogs are not included and there is a possibility that breeds with more health problems are more likely to be insured. Common for all the large databases is that the information recorded for each case and the details about the diagnostic workup can be sparse.

The reasoning behind calculating a geographically adjusted control group came from observations of different breed profiles at the two VTHs. SLU is situated in a middle-sized Swedish town, Uppsala, while NMBU is located in the city centre of Oslo, the capital of Norway. Registration numbers from different national kennel clubs reveal that breed distribution varies between countries. Even though the overall breed distribution is quite similar in Norway^7^ and Sweden,^8^ there are large regional variations (Additional file 1). Since both VTHs have a substantial number of referred patients, using the unadjusted registration numbers from the counties of Uppsala and Oslo, or the total numbers for each country, would create bias and not be representative of the actual source population. Adjusting the registration numbers from each of the counties by their relative contribution to the database of eligible cases, ensures this bias is kept at a minimum. The results from the logistic regression analysis (Table 5) show that the risk of becoming a case at NMBU is generally lower than at SLU. Since there are several other large small animal hospitals located near NMBU, while SLU is the largest hospital in Uppsala county, it is not surprising that the relative percentage of the control population seen at NMBU is smaller than at SLU.

Several limitations for this study must be acknowledged. Most importantly, only information from dogs examined at one of the participating VTHs were included. Therefore, information regarding dogs that were referred to other veterinary hospitals in the areas and for dogs whose owners did not pursue surgical treatment at the participating VTHs is lost. It is not unlikely that the treatment and referral strategies of dogs with the same orthopaedic disease might differ between breeds due to factors such as the complexity of the surgical procedure, size and temperament. It is therefore feasible that referral caseloads show a selection bias towards more complicated cases. For example, it is possible that small breed dogs with CCLD are underrepresented in our material because a substantial percentage of these dogs were treated conservatively or not referred in the first place. The information in the database cannot be retrospectively confirmed or rejected; therefore, all results rely on correct reporting of data. While ID-marking is mandatory for all Swedish dogs and for pure-breed Norwegian dogs to be registered in the national kennel club, it is voluntary for mixed-breed dogs in Norway. This discrepancy is a potential selection bias in the control group. However, the general Swedish and Norwegian dog populations are quite similar, and this is most likely true for mixed-breed dogs as well as pure-breeds. Moreover, a variety of cross-breeds (poodle mixes) have gained popularity over the last decades and are bred by breeders in a similar manner as pure-bred dogs. In addition, stray and shelter dogs are uncommon in Scandinavia; most dogs belong to an owner. Since the percentage of mixed-breed dogs in the Norwegian control population was higher than in the Swedish (Table 4), and with the aforementioned factors in mind, we believe the difference in ID-marking policy between Norway and Sweden to be of minor importance to our results. In addition, the control groups have been calculated separately for each country, and the logistic regression model adjusted for hospital.

Even though studies comparing the use of different control populations are available in the human literature [7], veterinary studies are lacking. The implications of using different control groups (i.e. hospital controls, insurance data, adjusted and unadjusted ID-registry data) in relation to breed susceptibility for disease should be addressed in future studies.

## Conclusions

Most of the results in the current study are in agreement with earlier reported breed predispositions for ED, MPL and CCLD, but in contrast to several other studies, an increased risk of CCLD was not identified for the Labrador retriever. The Staffordshire bull terrier was found to have an increased risk of ED. Although the country-specific results were mostly in concordance with each other, some discrepancies were noted. These findings highlight the importance of using large caseloads from different geographical regions and appropriate control groups when reporting breed susceptibility for disease.

## Additional file

**Additional file 1.** Control population calculations and raw registration numbers.

## Abbreviations

CCLD: cranial cruciate ligament disease
CKCS: Cavalier king Charles spaniel
ED: elbow dysplasia
GSD: German shepherd dog
MCD: medial compartment disease
MPL: medial patellar luxation
NMBU: Norwegian University of Life Sciences
OC: osteochondrosis
SLU: Swedish University of Agricultural Sciences
UAP: ununited anconeal process
VTH: Veterinary Teaching Hospital

## Footnotes

1 Regulation (EU) No 576/2013 of the European Parliament and of the Council of 12 June 2013 on the non-commercial movement of pet animals and repealing Regulation (EC) No 998/2003. https://eur-lex.europa.eu/eli/reg/2013/576/oj. (Accessed 30 November 2018).

2 Lag (2007:1150) om tillsyn över hundar och Katter. https://www.riksdagen.se/sv/dokument-lagar/dokument/svensk-forfattningssamling/lag-20071150-om-tillsyn-over-hundar-och_sfs-2007-1150. (Accessed 30 November 2018).

3 https://www.nkk.no/dyreid/category1232.html. (Accessed 30 November 2018).

4 Registration statistics, Swedish board of agriculture. http://www.jordbruksverket.se/amnesomraden/djur/olikaslagsdjur/hundarochkatter/hundregistret/statistik.4.45fb0f14120a3316ad78000672.html. (Accessed 28 November 2018).

5 Norwegian Kennel Club registration data. https://www.nkk.no/statistikk/category1098.html. (Accessed 28 November 2018).

6 International Elbow Working Group. http://www.vet-iewg.org/about/. (Accessed 29 October 2018).

7 Top 25 popular dog breeds in Norway 2017. https://www.nkk.no/getfile.php/131972994-1517566048/Dokumenter/Om%20NKK/Organisasjonen/Statistikk/Topp%2025%20registrerte%20raser%20i%202017.pdf. (Accessed 30 November 2018).

8 Registreringsstatisik 2017. Available at https://www.skk.se/sv/om-skk/det-har-ar-skk/press/. (Accessed 30 November 2018).
