# Correction to: Breed susceptibility for common surgically treated orthopaedic diseases in 12 dog breeds

**DOI:** 10.1186/s13028-020-0507-8

**Published:** 2020-02-19

**Authors:** Gudrun Seeberg Boge, Elena Regine Moldal, Maria Dimopoulou, Eystein Skjerve, Annika Bergström

**Affiliations:** 1 Department of Companion Animal Clinical Sciences, Faculty of Veterinary Medicine, Norwegian University of Life Sciences, P.O. Box 369 sentrum, 0102 Oslo, Norway; 2 Department of Clinical Sciences, Swedish University of Agricultural Sciences, P.O. Box 7054, 750 07 Uppsala, Sweden; 3 Department of Food Safety and Infection Biology, Faculty of Veterinary Medicine, Norwegian University of Life Sciences, P.O. Box 369 sentrum, 0102 Oslo, Norway

## Correction to: Acta Vet Scand (2019) 61:19 10.1186/s13028-019-0454-4

After publication of our article [1] we were notified that due to an error in the excel formula used to summarise the control population, the ID-registry data from the Swedish county Gävleborg was not included in the calculations. When including Gävleborg, as intended, the numbers in the adjusted Swedish control population change slightly. It does not influence the Norwegian control population.

The correct Tables 4 and 5, as well as the correct version of Additional file 1 are included in this correction.

**Table 4:** Breed distribution of dogs surgically treated for orthopaedic diseases and a geographically adjusted control group

Breed	Eligible cases			Control population		
	Sweden	Norway	Combined	Sweden	Norway	Combined
	N (%)	N (%)	N (%)	N (%)	N (%)	N (%)
Mixed-breed	114 (44.9)	53 (30.1)	167 (38.8)	3146 (28.0)	4359 (37.4)	7505 (32.5)
Border collie	8 (3.2)	5 (2.8)	13 (3.0)	331 (3.0)	425 (3.7)	756 (3.2)
CKCS	5 (2.0)	9 (5.1)	14 (3.3)	556 (5.0)	556 (4.8)	1112 (4.8)
Chihuahua	14 (5.5)	18 (10.2)	32 (7.4)	1113 (10.0)	985 (8.5)	2099 (9.0)
English cocker spaniel	1 (0.4)	3 (1.7)	4 (0.9)	547 (4.9)	539 (4.6)	1085 (4.7)
Flat-coated retriever	3 (1.2)	4 (2.3)	7 (1.6)	479 (4.3)	356 (3.1)	835 (3.5)
German shepherd dog	12 (4.7)	7 (4.0)	19 (4.4)	1125 (10.0)	591 (5.1)	1716 (7.3)
Golden retriever	17 (6.7)	9 (5.1)	26 (6.1)	1080 (9.6)	754 (6.5)	1834 (7.8)
Jack Russel terrier	9 (3.5)	6 (3.4)	15 (3.5)	457 (4.1)	710 (6.1)	1168 (5.1)
Labrador retriever	32 (12.6)	20 (11.4)	52 (12.1)	1345 (12.0)	823 (7.1)	2168 (9.3)
Rottweiler	19 (7.5)	22 (12.5)	41 (9.5)	540 (4.8)	469 (4.0)	1009 (4.3)
Shetland sheepdog	8 (3.2)	7 (4.0)	15 (3.5)	506 (4.5)	319 (2.7)	825 (3.5)
Staff. bull terrier	12 (5.8)	13 (7.4)	25 (5.8)	418 (3.7)	763 (6.6)	1181 (5.1)
Total (BuS)*	254 (51.2)	176 (36.1)	430 (43.7)	11,226 (100.0)	10,886 (100.0)	22,112 (100.0)
Other breeds*	242 (48.8)	312 (63.9)	554 (56.3)			
Total	496 (100.0)	488 (100.0)	984 (100.0)			

Control population calculated from registration numbers of each breed in the national ID-registries adjusted to reflect the source population of dogs surgically treated for orthopaedic diseases at the University Animal Hospital, Swedish University of Agricultural Sciences and the University Animal Hospital, Norwegian University of Life Sciences over a 5-year period

*BuS* breeds under study, *CKCS* Cavalier king Charles spaniel

*Data presented as number of dogs (percentage) of breeds under study

**Table 5:** Results from the logistic regression analyses of breed susceptibility for four common orthopaedic diseases in dogs

a) Elbow dysplasia												
Breed	SLU				NMBU				Combined			
	N (%)	OR	95% CI	P	N (%)	OR	95% CI	P	N (%)	OR	95% CI	P
Mixed-breed	13 (22.0)	1.00	Ref.	–	2 (7.41)	1.00	Ref.	–	15 (17.4)	1.00	Ref.	–
Flat-coated retriever	0 (0.0)	–	–	–	2 (7.41)	12.24	(1.72–87.18)	0.012	2 (2.3)	1.10	(0.25–4.83)	0.899
German shepherd dog	10 (17.0)	2.15	(0.94–4.92)	0.070	3 (11.11)	11.06	(1.84–66.35)	0.009	13 (15.1)	3.33	(1.57–7.62)	0.002
Golden retriever	7 (11.9)	1.57	(0.62–3.94)	0.338	2 (7.41)	5.78	(0.81–41.10)	0.080	9 (10.5)	2.23	(0.97–5.13)	0.058
Labrador retriever	19 (32.2)	3.42	(1.68–6.94)	0.001	9 (33.33)	23.83	(5.14–110.51)	< 0.001	28 (32.6)	5.79	(3.07–10.91)	< 0.001
Rottweiler	4 (6.8)	1.79	(0.58–5.52)	0.309	8 (29.63)	37.18	(7.87–175.58)	< 0.001	12 (14.0)	5.59	(2.60–11.99)	< 0.001
Staff. bull terrier	6 (10.2)	2.80	(1.06–7.41)	0.038	1 (3.7)	2.86	(0.25–31.54)	0.392	7 (8.1)	2.75	(1.12–6.77)	0.027
SLU	59 (68.9)								59 (45.0)	1.00	Ref.	–
NMBU					27 (60.0)				27 (20.6)	0.56	(0.35–0.90)	0.015
Other breeds	27 (31.1)				18 (40.0)				45 (34.4)			
Total	86 (100.0)				45 (100.0)				131 (100.0)			

b) Cranial cruciate ligament disease												
Breed	SLU				NMBU				Combined			
	N (%)	OR	95% CI	P	N (%)	OR	95% CI	P	N (%)	OR	95% CI	P
Mixed-breed	32 (44.4)	1.00	Ref.	–	13 (26.0)	1.00	Ref.	–	45 (36.9)	1.00	Ref.	–
Border collie	0 (0.0)	–	–	–	1 (2.0)	0.79	(0.10–6.05)	0.820	1 (0.8)	0.22	(0.03–1.59)	0.133
CKCS	0 (0.0)	–	–	–	2 (4.0)	1.21	(0.27–5.36)	0.805	2 (1.6)	0.29	(0.07–1.20)	0.087
Chihuahua	1 (1.39)	0.09	(0.01–0.65)	0.017	1 (2.0)	0.34	(0.04–2.61)	0.299	2 (1.6)	0.15	(0.04–0.63)	0.009
English cocker spaniel	0 (0.0)	–	–	–	1 (2.0)	0.62	(0.08–4.76)	0.648	1 (0.8)	0.15	(0.02–1.08)	0.059
German shepherd dog	0 (0.0)	–	–	–	1 (2.0)	0.57	(0.07–4.34)	0.585	1 (0.8)	0.09	(0.01–0.64)	0.016
Golden retriever	7 (9.7)	0.64	(0.28–1.45)	0.280	5 (10.0)	2.22	(2.22–1.17)	0.130	12 (9.8)	1.02	(0.54–1.93)	0.959
Jack russel terrier	7 (9.7)	1.51	(0.66–3.43)	0.330	2 (4.0)	0.94	(0.21–4.19)	0.940	9 (7.4)	1.30	(0.63–2.67)	0.473
Labrador retriever	6 (8.3)	0.44	(0.18–1.05)	0.065	7 (14.0)	2.85	(1.13–7.17)	0.026	13 (10.7)	0.92	(0.49–1.72)	0.795
Rottweiler	12 (16.7)	2.18	(1.12–4.27)	0.022	12 (24.0)	8.58	(3.89–18.91)	< 0.001	24 (19.7)	3.78	(2.29–6.25)	< 0.001
Shetland sheepdog	3 (4.17)	0.58	(0.18–1.91)	0.373	0 (0.0)	–	–	–	3 (2.5)	0.56	(0.17–1.81)	0.332
Staff. bull terrier	4 (5.6)	0.76	(0.27–2.16)	0.605	5 (10.0)	2.20	(0.78–6.18)	0.136	9 (7.4)	1.18	(0.58–2.42)	0.651
SLU	72 (50.3)								72 (27.7)	1.00	Ref.	–
NMBU					50 (42.7)				50 (19.2)	0.65	(0.45–0.94)	0.023
Other breeds	71 (49.7)				67 (57.3)				138 (53.1)			
Total	143 (100.0)				117 (100.0)				260 (100.0)			

c) Medial patellar luxation												
Breed	SLU				NMBU				Combined			
	N (%)	OR	95% CI	P	N (%)	OR	95% CI	P	N (%)	OR	95% CI	P
Mixed–breed	13 (50.0)	1.00	Ref.	–	8 (38.1)	1.00	Ref.	–	21 (44.7)	1.00	Ref.	–
CKCS	1 (3.9)	0.44	(0.06–3.33)	0.423	3 (14.3)	2.94	(0.78–11.11)	0.112	4 (8.2)	1.25	(0.43–3.66)	0.681
Chihuahua	9 (34.6)	1.96	(0.83–4.59)	0.123	8 (38.1)	4.43	(1.66–11.82)	0.003	17 (36.2)	2.8	(1.47–5.32)	0.002
Jack russel terrier	1 (3.9)	0.53	(0.07–4.06)	0.541	0 (0.0)	–	–	–	1 (2.1)	0.31	(0.42–2.30)	0.252
Shetland sheepdog	2 (7.7)	0.96	(0.22–4.25)	0.953	0 (0.0)	–	–	–	2 (4.3)	0.81	(0.19–3.49)	0.782
Staff. bull terrier	0 (0.0)	–	–	–	2 (9.5)	1.43	(0.30–6.74)	0.652	2 (4.3)	0.56	(0.13–2.40)	0.435
SLU	26 (36.1)								26 (19.8)	1.00	Ref.	–
NMBU					21 (35.6)				21 (16.0)	0.72	(0.40–1.29)	0.272
Other breeds	46 (63.9)				38 (64.4)				84 (64.1)			
Total	72 (100.0)				59 (100.0)				131 (100.0)			

d) Fractures of the radius and ulna												
Breed	SLU				NMBU				Combined			
	N (%)	OR	95% CI	P	N (%)	OR	95% CI	P	N (%)	OR	95% CI	P
Mixed–breed	19 (82.6)	1.00	Ref.	–	9 (52.9)	1.00	Ref.	–	28 (70.0)	1.00	Ref.	–
CKCS	1 (4.4)	0.30	(0.04–2.23)	0.238	0 (0.0)	–	–	–	1 (2.5)	0.23	(0.31–1.71)	0.151
Chihuahua	3 (13.0)	0.45	(0.13–1.51)	0.195	6 (35.3)	2.95	(1.05–8.31)	0.041	9 (22.5)	1.09	(0.51–2.32)	0.822
Shetland sheepdog	0 (0.0)	–	–	–	2 (11.8)	3.04	(0.65–14.11)	0.156	2 (5.0)	0.59	(0.14–2.51)	0.478
SLU	23 (48.9)								23 (20.2)	1.00	Ref.	–
NMBU					17 (25.4)				17 (14.9)	0.62	(0.33–1.16)	0.132
Other breeds	24 (51.1)				50 (74.6)				74 (64.9)			
Total	47 (100.0)				67 (100.0)				114 (100.0)			

Results from county-stratified and combined logistic regression analyses presented as Odds ratios (OR) and 95% confidence intervals (CIs). Breeds without cases of the disease in question were omitted

*CKCS* Cavalier king Charles spaniel, *SLU* Swedish University of Agricultural Sciences, *NMBU* Norwegian University of Life Sciences, *ref* referent category

In Table 4, the modified values are found in the Control population header, under the Sweden and Combined columns. In Table 5, the SLU and Combined columns contain the modified values.

## Supplementary information

**Additional file 1.** Control population calculations and raw registration numbers.
